# Immunomodulatory Effect of *H. Pylori CagA* Genotype and Gastric Hormones On Gastric Versus Inflammatory Cells Fas Gene Expression in Iraqi Patients with Gastroduodenal Disorders

**DOI:** 10.3889/oamjms.2016.032

**Published:** 2016-03-15

**Authors:** Ali Ibrahim Ali AL-Ezzy

**Affiliations:** Department of Pathology, College of Veterinary Medicine, Diyala University, Baquba, Diyala Province, Iraq

**Keywords:** Fas gene, gastric disorders, pepsinogens, gastrin -17, *H. pylori*, *CagA*

## Abstract

**AIM:**

To evaluate the Immunomodulatory effects of *CagA* expression; pepsinogen I, II & gastrin-17 on PMNs and lymphocytes Fas expression in inflammatory and gastric cells; demographic distribution of Fas molecule in gastric tissue and inflammatory cells.

**METHODS:**

Gastroduodenal biopsies were taken from 80 patients for histopathology and *H. pylori* diagnosis. Serum samples were used for evaluation of pepsinogen I (PGI); (PGII); gastrin-17 (G-17).

**RESULTS:**

Significant difference (p < 0.001) in lymphocytes & PMNs Fas expression; epithelial & lamina propria Fas localization among *H. pylori* associated gastric disorders. No correlation between grade of lymphocytes & PMNs Fas expression in gastric epithelia; lamina propria and types of gastric disorder. Significant difference (p < 0.001) in total gastric Fas expression, epithelial Fas; lamina propria and gastric gland Fas expression according to *CagA*, PGI; PGII; PGI/PGII; Gastrin-17. Total gastric Fas expression has significant correlation with *CagA*, PGII levels. Gastric epithelial and gastric lamina propria Fas expression have significant correlation with *CagA*, PGI; PGII levels. Significant difference (p < 0.001) was found in lymphocytes & PMNs Fas expression; epithelial & lamina propria localization of lymphocytes & PMNs Fas expression according to *CagA*, PGI; PGII; PGI/PGII; Gastrin-17. Lymphocytes Fas expression have correlation with PGI, PGII, PGI/PGII. PMNs Fas expression have correlation with PGI, PGII.

**CONCLUSION:**

Fas gene expression and localization on gastric and inflammatory cells affected directly by *H. pylori CagA* and indirectly by gastric hormones. This contributes to progression of various gastric disorders according to severity of *CagA* induced gastric pathology and gastric hormones disturbance throughout the course of infection and disease.

## Introduction

Helicobacter pylori infection is associated with several benign and malignant human diseases. Most infected individuals remain asymptomatic. If untreated, infection lasts for decades. The prevalence of infection ranging between 50% in developed countries and 90% in developing countries [1].

*H. pylori* occupies a unique niche, extremely acidic environment [2]. The urease of *H. pylori* is essential for its colonization and survival at extremely low pH, to ensure cytoplasmic homeostasis during large pH changes that occur during feeding. *H. pylori* can use molecular hydrogen as energy source; thus, its growth depends to some extent on the hydrogen excreted [3]. *H. pylori* infected gastric mucosa evolves through stages of chronic gastritis, intestinal metaplasia, glandular atrophy, and dysplasia before carcinoma develops.

*H. pylori* Infection is associated mostly with chronic antral gastritis, characterized by a mucosal infiltration of polymorphonuclear (PMNs) and mononuclear leukocytes [4]. Some studies have reported that *H. pylori* infection is suggested by the presence of active inflammation [3]. Neutrophil infiltration is a hallmark of active inflammation [4]. It is unknown whether neutrophil infiltration may be a marker of H. pylori infection.

It is now established that *Helicobacter pylori* causes more than 80% of duodenal ulcers and up to 60% of gastric ulcers [3]. The link between *Helicobacter pylori* infection and subsequent gastritis and peptic ulcer disease has been established through studies of human volunteers, antibiotic treatment studies and epidemiological studies. In some individuals, *Helicobacter pylori* also infect the corpus region of the stomach. This results in a more widespread inflammation that predisposes not only to ulcer in the corpus region, but also to stomach cancer [3].

Because of extremely low pH, the stomach is a hostile environment to most other microorganisms. The ability of *H. pylori* to flourish in the stomach has been attributed to protective mechanisms such as its production of urease, protecting the bacterium from gastric acidity by creating a basic microenvironment [5]. However, we reasoned that *H. pylori* might have evolved away to gain growth advantage in this niche, possibly by exploiting a gastric factor. A logical candidate would be one up regulated by *H. pylori* infection.

One such factor is the gastric hormone gastrin. Gastrin is produced as a prohormone by G cells located within the gastric antrum. The prohormone processed to shorter peptides, the most abundant of which is 17 amino acids long, termed gastrin-17 (G17). The major role attributed to gastrin within gastric tissue is the regulation of acid secretion [6]. After infection, gastrin levels are found to be consistently elevated and normal physiological negative feedback control of secretion is lost. Furthermore, after *H. pylori* eradication, gastrin levels are reduced and normal feedback control of gastrin secretion is restored [6, 7].

A number of studies have investigated the pathogenicity of *H. pylori* in relation to cytotoxic products, including urease, Cag, and vacuolating toxin (VacA) [8]. Potential apoptosis-inducing activity was reported in VacA [8] and urease [9]. Apoptosis in *H. pylori*-associated gastritis accompanies the activation of Fas and the Fas ligand system [10] in epithelial cells. Fas is a member of the tumor necrosis factor receptor family, which, when bound by its ligand, activates caspase-8, an initiator of the downstream apoptotic process that includes the cleavage of other death substrates, cellular and nuclear morphological changes and, ultimately, cell death [11]. Variations in host responses might cause the *H. pylori* mediated pathogenesis to result in a variety of clinical outcomes.

The aim of the present study is to evaluate the Immunomodulatory effects of *CagA* gene expression and gastric secretions (pepsinogen I, -II, Gastrin17) on inflammatory response mainly PMNs and lymphocytes infiltrations as well as expression of Fas gene in inflammatory and gastric cells under influence of *CagA* and gastric hormones and demographic distribution of Fas molecule in gastric tissue and inflammatory cells.

## Materials and Methods

### 

#### Patients

In this cross sectional study, (80) patients, age range 16-80 years, mean (47.24 ± 18.82) years, with clinical indications for upper gastrointestinal tract endoscopy during June 2013 to January 2015 were studied. Males represent 44 (55%) versus 36 (45%) females. This study was conducted according to the principles of Helsinki declaration. Before endoscopy, a full explanation about the purpose of this study to all patients was done. Dully-filled consent form obtained from all patients that agree to participate in the study. Approval of ethical review Committee of College of medicine – Diyala University - Iraq, was taken prior to initiation of the work at gastroenterology department of Baqubah teaching hospital in Diyala province - Iraq. Any patient under antibiotics or colloidal bismuth compounds for past one month treatment; having a history of previous gastric surgery and recent or active gastrointestinal bleeding was excluded from this study.

#### Methods

After topical pharyngeal anesthesia for overnight fasted Patients, A sterile flexible endoscope was introduced for full investigation of Stomach and duodenum. Six biopsy samples from congested, inflamed or erosive lesions were picked via sterile biopsy forceps. Samples were placed in Serim^®^ PyloriTek^®^ Test Kit for detection of urease activity. Each PyloriTek strip has a built-in positive analyte control and a negative control, which run concurrently with the test specimen. The PyloriTek positive control automatically appears with every test within the normal 1-hour time. With competitive tests the positive control is run after waiting 24 hours then inserting a urease positive control material [12].

Biopsy sample was placed in sterile glass slide with a drop of normal saline and teased with sterile scalpel to make smaller fragments of tissue then another sterile glass slide was placed over the teased first tissue and the tissue was crushed between the two glasses then stain by Gram’s staining. Existence of Gram negative spiral bacteria embedded in the tissue cells was diagnostic for *H. pylori* [13].

True positive results were considered if a combination of urease test and Gram stain give positive results for a single biopsy specimen [14].

Human Fas gene and *H. pylori* *CagA* gene expression detected by insitu hybridization procedure in 5µm thickness serial gastric mucosal sections fixed on positively charged slides using biotinylated long DNA probe for Human Fas gene; Cat. No. IH-60047 (fas-6001-B); *H. pylori*/*CagA* gene, Cat. No. IH-60061 (HPY-6001-B) (Maxim biotech-USA) and the DNA Probe hybridization/Detection System – In Situ Kit (Maxim biotech-USA), according to Maxim biotech instruction manual [15]. The examination and scoring were done under light microscope by pathologists at power X 400 according to the scoring system [16]. For serological assay blood was drawn from each patient during the visit to the endoscopy unit. Separated serum samples were stored at 27°C until analyses. Serum pepsinogen I(PGI) and II(PGII) and gastrin-17 (G-17) were assayed with ELISA using monoclonal antibodies to pepsinogen I and II and gastrin-17 (Biohit Diagnostics, Biohit, Devon, UK). All procedures were carried out according to the manufacturer’s instructions and results of pepsinogen I and II reported in µg/l and pmol/l for gastrin-17. The pepsinogen I: II ratio was calculated and reported in fraction [17].

#### Statistical analysis

Frequency of variables express as percentage. PG I, II and G-17 values express as mean ± standard deviation (Mean ± SD). Pearson test for correlation was used for non-categorical data. Chi-test used to compare the PG I, PGII, and G17 according to *CagA* gene expression.

The level of significance was 0.05 (two-tail) in all statistical testing; significant of correlations (Pearson, spearman) include also 0.01 (two-tail). Statistical analysis was performed using SPSS for windows TM version 17.0, and Microsoft Excel for windows 2010.

## Results

**Figure 1:**
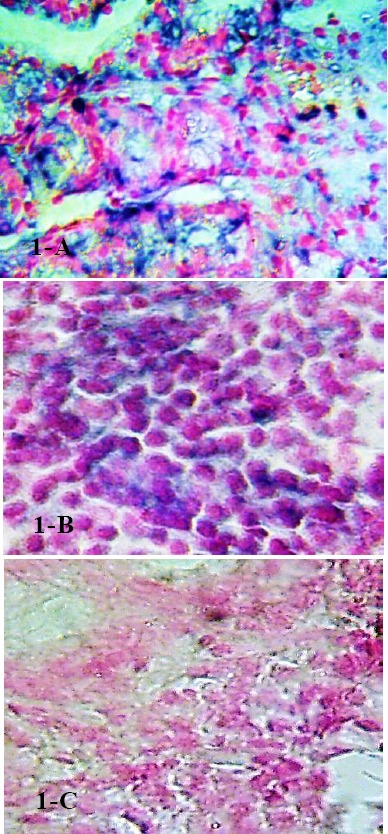
In situ hybridization for human Fas in gastric tissue section mainly in inflammatory cell infiltrates (A) lymphocytes; (B) PMNS, (C) negative expression. Bar size = 50 µm. staining by BCIP/NBT (bluish purple) counterstained with nuclear fast red

As shown in Table 1 gastric infiltrated lymphocytes have mild Fas expression in (61.25%) versus (31.25%) moderate expression (Figure 1). Mild and moderate lymphocytes Fas expression reported mainly in gastritis (25%) and lastly in, duodenitis (3.75%) and prepyloric ulcer (2.5%). Significant difference reported (p < 0.001) in lymphocytes Fas expression without correlation between grade of Fas expression and types of gastric disorder (p = 0.266). Gastric infiltrated PMNs have mild Fas gene expression in (46.25%), mainly in gastritis (12.5%) and lastly in prepyloric ulcer (2.5%) (Figure 1). Moderate PMNS Fas expression reported in (3.75%) in gastric ulcer and gastritis. Significant difference reported (p < 0.001) in PMNs Fas expression without correlation between grade of Fas expression and types of gastric disorder (p = 0.643).

**Table 1:** Lymphocytes and PMNs Fas gene expression and localization in gastroduodenal disorders

Presentation	Lymphocyte Fas gene					χ^2^	P value	R	P value

	<5%	5-25%	26-50%	50% >	Total				
Gastric ulcer	1 (1.25%)	10 (12.5%)	4 (5%)	0 (0%)	15 (18.75%)	197.467	0.000	0.126	0.266

Du	0 (0%)	6 (7.5%)	6 (7.5%)	0 (0%)	12 (15%)				

Gastropathy	3 (3.75%)	10 (12.5%)	2 (2.5%)	0 (0%)	15 (18.75%)				

Gastritis	2 (2.5%)	20 (25%)	8 (10%)	0 (0%)	30 (37.5%)				

Duodenitis	0 (0%)	3 (3.75%)	3 (3.75%)	0 (0%)	6 (7.5%)				

Prepyloric ulcer	0 (0%)	0 (0%)	2 (2.5%)	0 (0%)	2 (2.5%)				

Total	6 (7.5%)	49 (61.25%)	25 (31.25%)	0 (0%)	80 (100%)				

Presentation	*PMNs Fas gene*								

Gastric ulcer	6 (7.5%)	7 (8.75%)	2 (2.5%)	0 (0%)	15 (18.75%)	147.179	0.000	-0.053	0.643

Du	5 (6.25%)	7 (8.75%)	0 (0%)	0 (0%)	12 (15%)				

Gastropathy	9 (11.25%)	6 (7.5%)	0 (0%)	0 (0%)	15 (18.75%)				

Gastritis	19 (23.75%)	10 (12.5%)	1 (1.25%)	0 (0%)	30 (37.5%)				

Duodenitis	1 (1.25%)	5 (6.25%)	0 (0%)	0 (0%)	6 (7.5%)				

Prepyloric ulcer	0 (0%)	2 (2.5%)	0 (0%)	0 (0%)	2 (2.5%)				

Total	40 (50%)	37 (46.25%)	3 (3.75%)	0 (0%)	80 (100%)				

Presentation	Cellular Fas gene expressed in epithelial lining					χ^2^	P value	R	P value

	<5%	5-25%	26-50%	50% >	Total				

Gastric ulcer	0 (0%)	11 (13.75%)	4 (5%)	0 (0%)	15 (18.75%)	162.347	0.000	0.036	0.754

Du	0 (0%)	4 (5%)	8 (10%)	0 (0%)	12 (15%)				

Gastropathy	0 (0%)	9 (11.25%)	6 (7.5%)	0 (0%)	15 (18.75%)				

Gastritis	0 (0%)	14 (17.5%)	16 (20%)	0 (0%)	30 (37.5%)				

Duodenitis	0 (0%)	6 (7.5%)	0 (0%)	0 (0%)	6 (7.5%)				

Prepyloric ulcer	0 (0%)	0 (0%)	2 (2.5%)	0 (0%)	2 (2.5%)				

Total	0 (0%)	44 (55%)	36 (45%)	0 (0%)	80 (100%)				

Presentation	Cellular Fas gene expressed in lamina propria								

Gastric ulcer	0 (0%)	0 (0%)	0 (0%)	15 (18.75%)	15 (18.75%)	162.347	0.000	-0.036	0.754

Du	0 (0%)	0 (0%)	0 (0%)	12 (15%)	12 (15%)				

Gastropathy	0 (0%)	0 (0%)	0 (0%)	15 (18.75%)	15 (18.75%)				

Gastritis	0 (0%)	0 (0%)	0 (0%)	30 (37.5%)	30 (37.5%)				

Duodenitis	0 (0%)	0 (0%)	0 (0%)	6 (7.5%)	6 (7.5%)				

Prepyloric ulcer	0 (0%)	0 (0%)	0 (0%)	2 (2.5%)	2 (2.5%)				

Total	0 (0%)	0 (0%)	0 (0%)	80 (100%)	80 (100%)				

Mild (55%) to moderate (45%) Fas gene expression on inflammatory cells (lymphocytes and PMNs) infiltrated in gastric epithelial lining were reported. Mild Fas expression detected mainly in in gastritis (17.5%) and lastly in DU (5%). Moderate Fas expression detected in gastritis (20%), and finally in prepyloric ulcer (2.5%). There was significant difference (p < 0.001) in epithelial localization of lymphocytes & PMNs Fas among disorders without correlation (p = 0.754). Fas overexpression was detected in lymphocytes and PMNs infiltrated in lamina propria, mainly in gastritis (37.5%), gastric ulcer & gastropathy (18.75%), prepyloric ulcer (2.5%). There was significant difference (p < 0.001) in lamina propria localization of Fas expressing lymphocytes & PMNs among disorders without correlation (p = 0.754).

**Figure 2:**
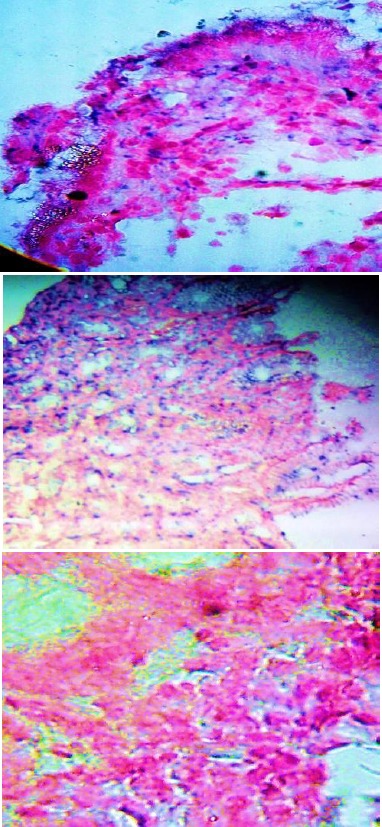
In situ hybridization for CagA Positive H. pylori in gastric tissue section-staining by BCIP/NBT (bluish purple) counterstained with nuclear fast red. Bar size=50 µm. A) Gastric epithelia, B) CagA expression extended to gastric pits, C) negative expression

As shown in Table 2, overexpression of Fas on gastric cells reported in (61.25%), moderate expression (31.25%), mild expression (5%). A sum of (58.75%) of gastroduodenal disorders infected with *CagA* positive *H. pylori* (Figure 2), (43.75%) have gastric Fas overexpression. Significant difference in Fas expression among disorders correlated with between *CagA* expression (p value < 0.001, p value = 0.001).

**Table 2:** Correlation of Gastric cells Fas gene expression and gastric secretion

Parameters		Gastric cells Fas gene					P value	R	P value

		<5%	5-25%	26-50%	50% >	Total			
*CagA* genotype	Positive	0(0%)	4 (5%)	8 (10%)	35 (43.75%)	47 (58.75%)	60.363	0.000	0.350	0.001

	Negative	2 (2.5%)	0 (0%)	17 (21.25%)	14 (17.5%)	33 (41.25%)				

*PGI*	<30 µg/L	0 (0%)	0 (0%)	0 (0%)	6 (7.5%)	6 (7.5%)	1149.995	0.000	-0.053	0.643

	30-160 µg/L	2 (2.5%)	0 (0%)	22 (27.5%)	25 (31.25%)	49 (61.25%)				

	*#x003E;160 µg/L	0 (0%)	4 (5%)	3 (3.75%)	18 (22.5%)	25 (31.25%)				

PGII	<3 µg/L	0 (0%)	0 (0%)	0 (0%)	0 (0%)	0 (0%)	1088.09	0.000	0.285	0.01

	3-15 µg/L	0 (0%)	0 (0%)	7 (8.75%)	12 (15%)	19 (23.75%)				

	*#x003E;15 µg/L	2 (2.5%)	4 (5%)	18 (22.5%)	37 (46.25%)	61 (76.25%)				

PGI/PGII	<3 µg/L	2 (2.5%)	0 (0%)	9 (11.25%)	22 (27.5%)	33 (41.25%)	1833.79	0.000	-0.163	0.156

	3-20 µg/L	0 (0%)	4 (5%)	16 (20%)	27 (33.75%)	47 (58.75%)				

	*#x003E;20 µg/L	0 (0%)	0 (0%)	0 (0%)	0 (0%)	0 (0%)				

Gastrin-17	< 1 pmol/ml	0 (0%)	0 (0%)	0 (0%)	0 (0%)	0 (0%)	586.08	0.000	0.020	0.858

	1-7 pmol/ml	2 (2.5%)	4 (5%)	19 (23.75%)	45 (56.25%)	70 (87.5%)				

	*#x003E;7 pmol/ml	0 (0%)	0 (0%)	6 (7.5%)	4 (5%)	10 (12.5%)				

Total		2 (2.5%)	4 (5%)	25 (31.25%)	49 (61.25%)	80 (100%)

Fas over expression reported in (7.5%) gastric disorders with hypopepsinogenemia, (31.25%) cases with normal PGI; (22.5%) with PGI hypersecretion. Moderate Fas expression reported in (27.5%) cases with normal PGI and (3.75%) with PGI hypersecretion. Significant difference among disorders in grade of Fas expression (p value < 0.001) without correlation with serum level of PGI (p value = 0.643).

Fas over expression reported in (15%) gastric disorders with normal PGII level; (46.25%) of cases with hypersecretion of PGII. Significant difference among disorders in grade of Fas expression (p value < 0.001) with correlation with serum level of PGII (p value = 0.01).

Gastric Fas over expression reported in (27.5%) of cases with PGI/PGII hyposecretion; (33.75%) of normal PGI/PGII. Significant difference among disorders in grade of Fas expression (p value < 0.001) without correlation with serum level of PGI/PGII (p value = 0.156). Significant difference in grade of Fas gene expression (p value < 0.001) without correlation with gastrin17 level (p value = 0.858).

**Figure 3:**
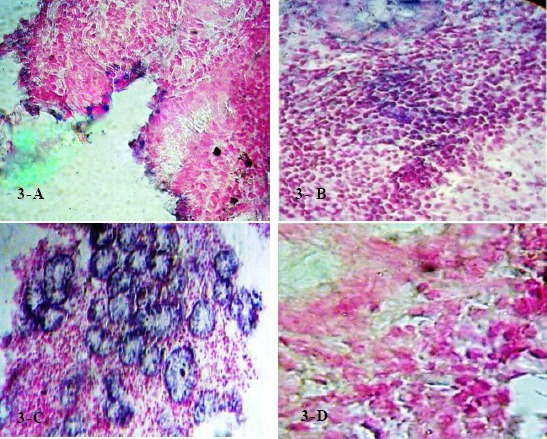
In situ hybridization for Fas gene in gastric tissue section. Staining by BCIP/NBT (bluish purple) counterstained with nuclear fast red. Bar size = 50 µm. A) positive Fas gene In situ hybridization expression in gastric epithelia; (B) positive Fas gene In situ hybridization expression in gastric lamina propria; (C) positive Fas gene In situ hybridization expression in gastric glands; (D) Negative In situ hybridization for Fas gene in gastric tissue section.

As in Table 3, gastric epithelia have mild Fas expression (61.25%) versus (38.75%) for moderate. *H. pylori* *CagA* infected gastric epithelia has moderate Fas expression in (26.25%) versus (32.5%) in mild with significant difference and correlation between *CagA* expression and grade of Fas expression (p value < 0.001, p value = 0.05) (Figure 3). Significant difference in grade of Fas gene expression (p value < 0.001) with correlation between serum level of PGI and Fas grade (p value < 0.001).

**Table 3:** Correlation of Gastric epithelial cells Fas gene expression and gastric secretion

Parameters		Gastric cells epithelial Fas gene					χ^2^	P value	R	P value

		<5%	5-25%	26-50%	50% >	Total				
*CagA* Genotype	Positive	0 (0%)	26 (32.5%)	21 (26.25%)	0 (0%)	47 (58.75%)	40.177	0.000	0.213 0.218*	0.058 0.052*

	Negative	0 (0%)	23 (28.75%)	10 (12.5%)	0 (0%)	33 (41.25%)				

PGI	<30 µg/l	0 (0%)	6 (7.5%)	0 (0%)	0 (0%)	6 (7.5%)	671.43	0.000	0.405	0.000

	30-160 µg/L	0 (0%)	34 (42.5%)	15 (18.75%)	0 (0%)	49 (61.25%)				

	>160 µg/L	0 (0%)	9 (11.25%)	16 (20%)	0 (0%)	25 (31.25%)				

PGII	<3 µg/l	0 (0%)	0 (0%)	0 (0%)	0 (0%)	0 (0%)	650.29	0.000	0.341	0.002

	3-15 µg/L	0 (0%)	15 (18.75%)	4 (5%)	0 (0%)	19 (23.75%)				

	>15 µg/L	0 (0%)	34 (42.5%)	27 (33.75%)	0 (0%)	61 (76.25%)				

PGI/PGII	<3 µg/l	0 (0%)	22 (27.5%)	11 (13.75%)	0 (0%)	33 (41.25%)	1044.64	0.000	0.079	0.487

	3-20 µg/L	0 (0%)	27 (33.75%)	20 (25%)	0 (0%)	47 (58.75%)				

	>20 µg/L	0 (0%)	0 (0%)	0 (0%)	0 (0%)	0 (0%)				

Gastrin-17	< 1 pmol/ml	0 (0%)	0 (0%)	0 (0%)	0 (0%)	0 (0%)	348.498	0.000	-0.075	0.508

	1-7 pmol/ml	0 (0%)	40 (50%)	30 (37.5%)	0 (0%)	70 (87.5%)				

	>7 pmol/ml	0 (0%)	9 (11.25%)	1 (1.25%)	0 (0%)	10 (12.5%)				

* Spearman Correlation

Significant difference in gastric epithelia Fas grade (p value < 0.001) with correlation between PGII levels (p value = 0.0002). Among (58.75%) patients of normal PGI/PGII, (25%) have moderate grade of gastric Fas expression, (33.75%) mild. Significant difference in grade of gastric epithelial Fas expression (p value < 0.001) without correlation between level of PGI/PGII and grade Fas expression (p value = 0.487). Normal Gastrin17 detected in (87.5%), (37.5%) have moderate Fas expression and (50%) was mild. Hypergastrinemia was detected in (12.5%), (1.25%) have moderate Fas expression, (11.25%) mild. Significant difference in grade of Fas expression (p value < 0.001) without correlation between gastrin17 and grade of Fas expression (p value = 0.508).

As shown in Table 4, mild Fas expression in lamina propria (56.25%) versus (43.75%) moderate. Significant difference and correlation between *CagA* expression and Fas expression in lamina propria (p value = 0.001, p value = 0.056) (Figure 3). Significant difference among disorders in grade of Fas expression (p value < 0.001) with significant correlation between serum PGI and grade of Fas expression in gastric lamina propria (p value = 0.05). Normal PGII level detected in (23.75%) patients, (15%) have moderate grade of Fas expression and (8.75%) was mild. Hypersecretion of PGII was determined in (76.25%) patients, (28.75%) have moderate grade of gastric Fas expression, (47.5%) was mild. Significant difference in grade of Fas expression (p value < 0.001) with correlation between PGII level and grade of Fas expression in gastric lamina propria (p value = 0.001). Significant difference in grade of Fas expression in gastric lamina propria (p value < 0.001) without correlation between serum PGI/PGII and grade of gastric lamina propria Fas expression (p value = 0.519). Significant difference in grade of Fas expression (p value < 0.001) without correlation between gastrin17 level and grade of gastric lamina propria Fas expression (p value = 0.575).

**Table 4:** Correlation of Gastric lamina propria, gastric glands Fas gene expression and gastric secretion

Parameters		Gastric Fas gene expressed in lamina propria					P value	r	P value
		<5%	5-25%	26-50%	50% >	Total		
Cag A genotype	Positive	0 (0%)	32 (40%)	15 (18.75%)	0 (0%)	47 (58.75%)	44.315	0.001	-0.215	0.056

	Negative	0 (0%)	13 (16.25%)	20 (25%)	0 (0%)	33 (41.25%)				

Pgi	<30 µg/l	0 (0%)	6 (7.5%)	0 (0%)	0 (0%)	6 (7.5%)	782.410	0.000	-.212	0.05

	30-160 µg/L	0 (0%)	19 (23.75%)	30 (37.5%)	0 (0%)	49 (61.25%)				

	>160 µg/L	0 (0%)	20 (25%)	5 (6.25%)	0 (0%)	25 (31.25%)				

Pgii	<3 µg/l	0 (0%)	0 (0%)	0 (0%)	0 (0%)	0 (0%)	746.31	0.000	-0.361	0.001

	3-15 µg/L	0 (0%)	7 (8.75%)	12 (15%)	0 (0%)	19 (23.75%)				

	>15 µg/L	0 (0%)	38 (47.5%)	23 (28.75%)	0 (0%)	61 (76.25%)				

Pgi/pgii	<3 µg/l	0 (0%)	19 (23.75%)	14 (17.5%)	0 (0%)	33 (41.25%)	1300.262	0.000	-0.073	0.519

	3-20 µg/L	0 (0%)	26 (32.5%)	21 (26.25%)	0 (0%)	47 (58.75%)				

	>20 µg/L	0 (0%)	0 (0%)	0 (0%)	0 (0%)	0 (0%)				

Gastrin17	< 1 pmol/ml	0 (0%)	0 (0%)	0 (0%)	0 (0%)	0 (0%)	444.840	0.000	-0.064	0.575

	1-7 pmol/ml	0 (0%)	40 (50%)	30 (37.5%)	0 (0%)	70 (87.5%)				

	>7 pmol/ml	0 (0%)	5 (6.25%)	5 (6.25%)	0 (0%)	10 (12.5%)

Parameters		Fas gene expressed in Gastric glands					X2	P value	r	P value

Cag A genotype	Positive	0 (0%)	2 (2.5%)	21 (26.25%)	24 (30%)	47 (58.75%)	42.496	0.000	0.037	0.743

	Negative	0 (0%)	2 (2.5%)	12 (15%)	19 (23.75%)	33 (41.25%)				

Pgi	<30 µg/l	0 (0%)	0 (0%)	0 (0%)	6 (7.5%)	6(7.5%)	723.02	0.000	-0.118	0.296

	30-160 µg/L	0 (0%)	4 (5%)	22 (27.5%)	23 (28.75%)	49 (61.25%)				

	>160 µg/L	0 (0%)	0 (0%)	11 (13.75%)	14 (17.5%)	25 (31.25%)				

Pgii	<3 µg/l	0 (0%)	0 (0%)	0 (0%)	0 (0%)	0 (0%)	673.75	0.000	0.08	0.501

	3-15 µg/L	0 (0%)	0 (0%)	7 (8.75%)	12 (15%)	19 (23.75%)				

	>15 µg/L	0 (0%)	4 (5%)	26 (32.5%)	31 (38.75%)	61 (76.25%)				

Pgi/pgii	<3 µg/l	0 (0%)	4 (5%)	5 (6.25%)	24 (30%)	33 (41.25%)	1067.56	0.000	0.008	0.947

	3-20 µg/L	0 (0%)	0 (0%)	28 (35%)	19 (23.75%)	47 (58.75%)				

	>20 µg/L	0 (0%)	0 (0%)	0 (0%)	0 (0%)	0 (0%)				

Gastrin17	< 1 pmol/ml	0 (0%)	0 (0%)	0 (0%)	0 (0%)	0 (0%)	363.42	0.000	0.121	0.286

	1-7 pmol/ml	0 (0%)	4 (5%)	30 (37.5%)	36 (45%)	70 (87.5%)				

	>7 pmol/ml	0 (0%)	0 (0%)	3 (3.75%)	7 (8.75%)	10 (12.5%)				

As shown in Table 4, Fas overexpression appear to be more frequent (53.75%) than moderate (41.25%) and mild (5%) grade of expression in gastric glands. Significant difference without correlation between *CagA* expression and grade of Fas in gastric glands (p value = 0.001, p value = 0.743).

Significant difference in grade of Fas expression (p value < 0.001) without significant correlation between serum PGI and grade of gastric glands Fas expression (p value = 0.296). Significant difference among disorders in grade of Fas (p value < 0.001) without correlation between serum PGII and grade of gastric glands Fas expression (p value = 0.501). Significant difference in grade of gastric gland Fas expression (p value < 0.001) without correlation between PGI/PGII serum level and grade of gastric cells Fas (p value = 0.947). Significant difference among disorders in grade of Fas gene expression (p value < 0.001) without correlation between gastrin17 and grade of gastric glands Fas expression (p value = 0.286).

As shown in Table 5, moderate lymphocytes Fas expression detected in (31.25%) versus (27.5%) mild among *CagA* positive cases. Significant difference without correlation between *CagA* expression and grade of Fas in lymphocytes (p value = 0.001, p value = 0.112). Significant difference in grade of Fas (p value < 0.001) with significant correlation between serum PGI and grade of lymphocytes Fas expression (p value = 0.009). Normal PGII level detected in (23.75%) of patients, (6.25%) have moderate and mild grades of lymphocytes Fas expression. Hypersecretion of PGII determined in (76.25%) of patients, (25%) have moderate grade of lymphocytes Fas, (55%) was mild. Significant difference in grade of lymphocytes Fas (p value < 0.001) with correlation between PGII levels and grade of gastric lymphocytes Fas (p value = 0.035). Significant difference in gastric lymphocytes Fas (p value < 0.001) with correlation between serum PGI/PGII and grade of gastric lymphocytes Fas expression (p value = 0.046).

**Table 5:** Correlation of Lymphocytes Fas gene and gastric secretion

Parameters		Lymphocyte Fas gene					χ^2^	P value	r	P value

		<5%	5-25%	26-50%	50% >	Total			
Cag A genotype	Positive	0 (0%)	22 (27.5%)	25 (31.25%)	0 (0%)	47 (58.75%)	51.219	0.000	0.179	0.112

	Negative	2 (2.5%)	27 (33.75%)	4 (5%)	0 (0%)	33 (41.25%)				

*PGI*	<30 µg/L	0 (0%)	5 (6.25%)	1 (1.25%)	0 (0%)	6 (7.5%)	763.23	0.000	-0.291	0.009

	30-160 µg/L	2 (2.5%)	28 (35%)	17 (21.25%)	0 (0%)	47 (58.75%)				

	>160 µg/L	4 (5%)	16 (20%)	7 (8.75%)	0 (0%)	27 (33.75%)				

PGII	<3 µg/L	0 (0%)	0 (0%)	0 (0%)	0 (0%)	0 (0%)	644.31	0.000	0.236	0.035

	3-15 µg/L	0 (0%)	5 (6.25%)	5 (6.25%)	0 (0%)	10 (12.5%)				

	>15 µg/L	6 (7.5%)	44 (55%)	20 (25%)	0 (0%)	70 (87.5%)				

PGI/PGII ratio	<3 µg/L	2 (2.5%)	18 (22.5%)	13 (16.25%)	0 (0%)	33 (41.25%)	1259.89	0.000	-0.224	0.046

	3-20 µg/L	4 (5%)	31 (38.75%)	12 (15%)	0 (0%)	47 (58.75%)				

	>20 µg/L	0 (0%)	0 (0%)	0 (0%)	0 (0%)	0 (0%)				

Gastrin17	< 1 pmol/ml	0 (0%)	0 (0%)	0 (0%)	0 (0%)	0 (0%)	494.53	0.000	-0.050	0.663

	1-7 pmol/ml	6 (7.5%)	39 (48.75%)	25 (31.25%)	0 (0%)	70 (87.5%)				

	>7 pmol/ml	0 (0%)	10 (12.5%)	0 (0%)	0 (0%)	10 (12.5%)

Parameters		PMNs Fas gene					χ^2^	P value	r	P value

Cag A genotype	Positive	20 (25%)	24 (30%)	3 (3.75%)	0 (0%)	47 (58.75%)	29.177	0.015	0.262	0.019

	Negative	23 (28.75%)	10 (12.5%)	0 (0%)	0 (0%)	33 (41.25%)				

*PGI*	<30 µg/L	3 (3.75%)	3 (3.75%)	0 (0%)	0 (0%)	6 (7.5%)	654.03	0.000	-0.296	0.008

	30-160 µg/L	21 (26.25%)	25 (31.25%)	3 (3.75%)	0 (0%)	49 (61.25%)				

	>160 µg/L	16 (20%)	9 (11.25%)	0 (0%)	0 (0%)	25 (31.25%)				

PGII	<3 µg/L	0 (0%)	0 (0%)	0 (0%)	0 (0%)	0 (0%)	534.59	0.000	-.018	0.874

	3-15 µg/L	9 (11.25%)	7 (8.75%)	3 (3.75%)	0 (0%)	19 (23.75%)				

	>15 µg/L	31 (38.75%)	30 (37.5%)	0 (0%)	0 (0%)	61 (76.25%)				

PGI/PGII	<3 µg/L	16 (20%)	17 (21.25%)	0 (0%)	0 (0%)	33 (41.25%)	1033.23	0.000	-0.014	0.905

	3-20 µg/L	24 (30%)	20 (25%)	3 (3.75%)	0 (0%)	47 (58.75%)				

	>20 µg/L	0 (0%)	0 (0%)	0 (0%)	0 (0%)	0 (0%)				

Gastrin17	< 1 pmol/ml	0 (0%)	0 (0%)	0 (0%)	0 (0%)	0 (0%)	324.95	0.000	-0.007	0.952

	1-7 pmol/ml	34 (42.5%)	33 (41.25 %)	3 (3.75%)	0 (0%)	70 (87.5 %)				

	>7 pmol/ml	6 (7.5 %)	4 (5%)	0 (0%)	0 (0%)	10 (12.5 %)				

Significant difference in grade of Fas expression (p value < 0.001) without correlation between level of gastrin17 and grade of gastric lymphocyte Fas expression (p value = 0.663). Significant difference with correlation between *CagA* expression and grade of PMNs Fas (p value = 0.015, p value = 0.019), (Table 5). Significant difference in grade of PMNs Fas (p value < 0.001) with significant correlation between level of PGI and PMNs Fas grade (p value = 0.008). Significant difference in grade of PMNs Fas (p value < 0.001) without correlation with PGII level (p value = 0.874). Significant difference in grade of PMNs Fas expression (p value < 0.001) without correlation between level of PGI/PGII and grade of PMNs Fas (p value = 0.905). Normal gastrin17 detected in (87.5%), (3.75%) have moderate PMNs Fas grade, (41.25 %) have mild PMNs Fas.

Hypergastrinemia detected in (12.5%), (5%) have mild grade of PMNs Fas. Significant difference in grade of PMNs Fas (p value < 0.001) without correlation between serum gastrin17 and grade of gastric glands Fas (p value = 0.952).

As shown in Table 6, moderate grade of PMNs & lymphocytes Fas in gastric epithelia reported in (27.5%) of cases, (31.25%) have mild expression. Significant difference without correlation between *CagA* and grade of PMNs & lymphocytes Fas expression (p value = 0.015, p value = 0.358). Significant difference among disorders in grade of PMNs &lymphocytes Fas expression (p value < 0.001) without significant correlation between PGI levels and grade of PMNs & lymphocytes Fas expression (p value = 0.737). Significant difference among disorders in grade of PMNs & lymphocytes Fas (p value < 0.001) without correlation with PGII levels (p value = 0.191). Hyposecretion of PGI/PGII detected in (41.25%), (21.25%) have mild grade of PMNs & lymphocytes Fas expression. Significant difference in grade of PMNs & lymphocytes Fas (p value < 0.001) without correlation (p value = 0.547). Significant difference among disorders in grade of PMNs Fas expression (p value < 0.001) without correlation between serum level of gastrin-17 and grade of PMNs & lymphocytes Fas expression (p value = 0.388).

**Table 6:** Correlation of cellular Fas gene and gastric secretion

Parameters		Cellular Fas gene expressed in epithelia					χ^2^	P value	r	P value

		<5%	5-25%	26-50%	50% >	Total				
*Cag A genotype*	Positive	0 (0%)	25 (31.25%)	22 (27.5%)	0 (0%)	47 (58.75%)	43.225	0.000	-0.104	0.358

	Negative	0 (0%)	19 (23.75%)	14 (17.5%)	0 (0%)	33 (41.25%)				

*PGI*	<30 µg/L	0 (0%)	4 (5%)	2 (2.5%)	0 (0%)	6 (7.5%)	762.552	0.000	0.038	0.737

	30-160 µg/L	0 (0%)	27 (33.75%)	22 (27.5%)	0 (0%)	49 (61.25%)				

	>160 µg/L	0 (0%)	13 (16.25%)	12 (15%)	0 (0%)	25 (31.25%)				

PGII	<3 µg/L	0 (0%)	0 (0%)	0 (0%)	0 (0%)	0 (0%)	689.201	0.000	0.148	0.191

	3-15 µg/L	0 (0%)	15 (18.75%)	4 (5%)	0 (0%)	19 (23.75%)				

	>15 µg/L	0 (0%)	29 (36.25%)	32 (40%)	0 (0%)	61 (76.25%)				

PGI/PGII	<3 µg/L	16 (20%)	17 (21.25%)	0 (0%)	0 (0%)	33 (41.25%)	1120.384	0.000	-0.068	0.547

	3-20 µg/L	24 (30%)	20 (25%)	3 (3.75%)	0 (0%)	47 (58.75%)				

	>20 µg/L	0 (0%)	0 (0%)	0 (0%)	0 (0%)	0 (0%)				

Gastrin17	< 1 pmol/ml	0 (0%)	0 (0%)	0 (0%)	0 (0%)	0 (0%)	341.613	0.000	-0.098	0.388

	1-7 pmol/ml	34 (42.5%)	33 (41.25%)	3 (3.75%)	0 (0%)	70 (87.5%)				

	>7 pmol/ml	6 (7.5%)	4 (5%)	0 (0%)	0 (0%)	10 (12.5%)				

Parameters		Cellular Fas gene expressed in lamina propria								

		<5%	5-25%	26-50%	50% >	Total	χ^2^	P value	r	P value

*Cag A genotype*	Positive	0 (0%)	32 (40%)	15 (20%)	0 (0%)	47 (58.75%)	44.395	0.001	-.215	0.056

	Negative	0 (0%)	13 (16.25%)	20 (25%)	0 (0%)	33 (41.25%)				

*PGI*	<30 µg/L	0 (0%)	3 (3.75%)	3 (3.75%)	0 (0%)	6 (7.5%)	762.552	0.000	-0.038	0.737

	30-160 µg/L	0 (0%)	22 (27.5%)	27 (33.75%)	0 (0%)	49 (61.25%)				

	>160 µg/L	0 (0%)	20 (25%)	5 (6.25%)	0 (0%)	25 (31.25%)				

PGII	<3 µg/L	0 (0%)	0 (0%)	0 (0%)	0 (0%)	0 (0%)	689.201	0.000	0.148	0.191

	3-15 µg/L	0 (0%)	0 (0%)	0 (0%)	19 (23.75%)	19 (23.75%)				

	>15 µg/L	0 (0%)	0 (0%)	0 (0%)	61 (76.25%)	61 (76.25%)				

PGI/PGII	<3 µg/L	0 (0%)	19 (23.75%)	14 (17.5%)	0 (0%)	33 (41.25%)	1120.384	0.000	0.068	0.547

	3-20 µg/L	0 (0%)	26 (32.5%)	21 (26.25%)	0 (0%)	47 (58.75%)				

	>20 µg/L	0 (0%)	0 (0%)	0 (0%)	0 (0%)	0 (0%)				

Gastrin17	< 1 pmol/ml	0 (0%)	0 (0%)	0 (0%)	0 (0%)	0 (0%)	586.079	0.000	0.020	0.858

	1-7 pmol/ml	0 (0%)	40 (50%)	30 (37.5%)	0 (0%)	70 (87.5%)				

	>7 pmol/ml	0 (0%)	5 (6.25%)	5 (6.25%)	0 (0%)	10 (12.5%)				

As shown in Table 6, significant difference with marginal correlation between *CagA* gene expression and grade of PMNs & lymphocytes Fas (p value = 0.001, p value = 0.056). Significant difference in grade of PMNs & lymphocytes Fas (p value < 0.001) without significant correlation between serum level of PGI and grade of PMNs &lymphocytes Fas (p value = 0.737). Significant difference among disorders in grade of PMNs & lymphocytes Fas (p value < 0.001) without correlation with PGII levels (p value = 0.191).

As shown in Table 6, significant difference among disorders in grade of PMNs & lymphocytes Fas expression in lamina propria (p value < 0.001) without correlation (p value = 0.547). Significant difference among disorders in grade of PMNs Fas gene (p value < 0.001) without correlation between level of gastrin-17 and grade of PMNs & lymphocytes Fas expression (p value = 0.858).

## Discussion

In this cross sectional study, (80) of *H. pylori* infected patients, age range 16-80 years, mean (47.24 ± 18.82) years were studied. The frequency of *H. pylori* associated disorders in current study starting from gastritis (37.5%), gastropathy & gastric ulcer (18.75%), DU(15%), duodenitis (7.5%), prepyloric ulcer (2.5%) which come in line with others[4].

In current study high grade of Fas gene expression was not detected at all. Mild lymphocytes expression (5-25%) reported in (61.25%), followed by moderate grade (26-50%) of expression (31.25%) among different disorders. In PMNs, mild Fas expression was reported in (46.25%) versus (3.75%) moderate the frequency of Fas gene expression more frequently reported in gastritis and gastropathy. Even there was significant difference in lymphocytes Fas expression among disorders, there was no correlation between this expression and type of gastric disorder. This is a logical finding because the induction of Fas expression via infiltrating lymphocytes and PMNs is associated and attributed to the induction via *H. pylori* infection [10]. Low Fas expression in infiltrating PMNs compared with infiltrating lymphocytes may attributed to the chronic active inflammatory reaction among different disorders [11].

One of interesting points that the lymphocytes and PMNs Fas expression in epithelial lining range from mild (gastric ulcer; gastritis; gastropathy) to moderate (gastritis; DU; gastropathy) with significant difference. While in lamina propria, infiltrating lymphocytes and PMNs among all disorders have high grade of Fas gene expression. This may attributed to the ability of *H. pylori* to invade deeply in gastric tissue gastric beyond mucosal epithelia, which indicates initiation of interaction between *H. pylori* and inflammatory cells in lamina propria leading to activation of NFkB gene in gastric as well as Lymphocytes and PMNs, finally IL8 production and increase oxidative stress in gastric tissue leading final to increase Fas gene production which is more obvious in lamina propria than in gastric epithelia, reflecting the underling pathology in near future [1, 10, 11].

In current study *CagA* genotype have a positive effect and correlation on the gastric cells Fas over expression in (43.75%). Mild expression detected in (10%). This results come in line with [1, 10]. This result come in concordance with opinion of [10, 18] stated that whenever *H. pylori* have potent Cag PAI, bacterial adhesion, good signals will be received by the gastric epithelial cells which reflect its response by increasing of expression of MHCII to play as antigen presenting cell (APC) and strong Th1 response will be occur with obvious IFNγ secretion which acts as good stimulator for up-regulation of FAS and even FASL in gastroduodenal tissue and tissue infiltrating lymphocytes (TILs).

One of exciting things in this study, the fluctuation of gastric Fas expression independently from a status of gastric hormones. All cases with hyposecretion of PGI; PGI/PGII characterized by gastric Fas overexpression, (7.5%) vs. (27.5%). On the other hand all cases with normal gastric hormone level also associated with gastric Fas overexpress, PGI (31.25%), PGII (15%), PGI/PGII (33.75%), G17 (56.25%) without any significant correlation between a status of PGI, PGI/PGII, Gastrin 17 and gastric Fas overexpression. PGII have positive correlation with gastric Fas overexpression among different disorders.

These results indicating a *H. pylori* induced pangastric type of mucosal inflammation even to duodenal region that reflects the disruption in gastric hormones secretion mainly over production of PGI (31.25%), PGII (76.25%), G17 (5%) [19]. Gastric Fas expression and gastric hormones have indirect correlation. Mucosal colonization by the *H. pylori* ignites a cascade of events that result in large increases in inflammatory cytokines in the infected tissue which were originated from the gastric mucosa as well as from infiltrating inflammatory cells. Among these inflammatory cytokines, IL-1, IL-2, TNF-α, and IFNγ that have been shown to up-regulate the expression of Fas antigen in gastric as well as inflammatory cells. T-helper type 1 (Th1) cells are selectively increased during *H. pylori* infection [20]. Th1 cytokines, such as IFNγ and TNF-α, can increase the release of proinflammatory cytokines, such as IL-8 from the epithelium as well as Fas and Fas ligand (FasL) 34. Furthermore, these cytokines can also increase the expression of MHC class II molecules by gastric epithelial cells, thereby increasing the binding of *H. pylori* to the gastric epithelium [21].

One of frustrating things that there was no clinical study to evaluates the correlation between gastric Fas expression and the level of gastric hormones. One of remarkable finding in current study when making topographical analysis for the correlation between gastric hormones and gastric Fas expression in gastric epithelia, lamina propria and gastric glands. This study reported a positive correlation between gastric Fas expression and PGI;PGII in gastric epithelia, lamina propria but not in gastric gland even with presence of significant difference in gastric hormones according to Fas gene expression.

Attachment of *H. pylori* on specific molecules expressed and acts as a receptor in the gastric epithelia, MHCII and CD74 using multiple adhesins that have been identified on the outer membrane of *H. pylori* such as BabA, SabA. BabA and SabA bind to fucosylated and sialylated blood group antigens, respectively. While the attachment of *H. pylori* using BabA as an adhesin does not appear to induce signaling or immune responses from host cells, SabA appears to be required for activation of neutrophils and the resulting oxidative burst by binding to sialylated neutrophil receptors [22]. This inflammatory signal extended throughout gastric epithelia to lamina propria also via the effect of neutrophil activating protein and urease as well as *CagA* production by *H. pylori* which in turn leads to activation of lymphocytes and gastric NFkB causing production of proinflammatory cytokines, mainly IL8 and hence the gastric epithelia and lamina propria sinking with inflammatory reaction and cells leading to increase of Fas expression on gastric epithelia and lamina propria as well as inflammatory cells [1, 11, 23]. Because *CagA* interacts with important signaling mediators in the gastric cells, it is considered responsible for changes in cell morphology, adhesion and turnover [23]. *CagA* cause increase in Fas expression independently or in conjunction with other virulence factors such as urease [10, 11]. At the same time as a results of pan gastric inflammatory reaction this leads to direct effects on PGII producing cells causing increase in PGII production and as a results of neuroendocrine activity through vagus nerve and gastrin, PGI also affected. Fas expression on gastric glands has no correlation with gastric hormones even in the presence of significant differences in hormones according to grade of Fas expression may be associated with fact that gastric glands appear to be Immunological privileged site.

In current study, *H. pylori* influence on apoptosis of lymphocytes in gastric mucosa lymphocytes Fas expression has positive correlation with PGI; PGII, PGI/PGII. Obvious fluctuation of lymphocytes Fas according to gastric hormones from mild to moderate expression as in Table 5. One of remarkable finding was the mild to moderate expression of Fas on PMNS which has positive correlation with PGI serum level only. This may attributed to chronic active pangastric inflammation affecting the PGI, PGII producing chief cells within gastric mucosa, reflecting by (3.75%) mild expression of PMNs, (6.25%) in lymphocytes and clinically patients suffered from atrophic corpus gastritis. The mild to moderate expression of Fas on gastric tissue infiltrating lymphocytes give an indication about the immunopathological role of these cells in underling clinical diseases by relatively low expression of apoptotic marker (Fas) in lymphocytes as well as PMNs this will leads to delay spontaneous apoptosis and prolong their survival. It results in the prolonged activity of cytokines secreted by these cells and therefore augments their damaging action upon gastric mucosa [24, 25]. On the other hand the mild to moderate expression of Fas receptors on lymphocytes and PMNs act in delay of their proliferation and affects its ability to get rid of *H. pylori* from gastric tissue, and at sum this will cause persistence infection and a protective strategy for *H. pylori* [24, 26].

When making topographical analysis for the correlation between gastric hormones and inflammatory cells (lymphocytes & PMNs) Fas expression in gastric epithelia, lamina propria; No correlation was reported between inflammatory cells (lymphocytes & PMNs) Fas expression and gastric hormones secretion in gastric epithelia, lamina propria even with presence of significant difference in gastric hormones according to Fas gene expression as in Table 6. This finding reflect the role of; and give an indication that *H. pylori* acts as inducer for Fas gene expression on gastric cells as well as lymphocytes & PMNs, directly through its virulence factors, mainly products of Cag pathogenicity island, *CagA* and others, and indirectly through cytokines produce by TH1 cells such as IFNγ as well as the oxidative stress produced by reactive oxygen species from inflammatory cells infiltrated into gastric tissue due to *H. pylori* infection [10, 11, 24].

In conclusion, Fas gene expression and localization on gastric and inflammatory cells affected directly by *H. pylori* *CagA* and indirectly by gastric hormones. This contributes to progression of various gastric disorders according to severity of *CagA* induced gastric pathology and gastric hormones disturbance throughout the course of infection and disease.
